# Annexins in Influenza Virus Replication and Pathogenesis

**DOI:** 10.3389/fphar.2018.01282

**Published:** 2018-11-15

**Authors:** Patrick Baah Ampomah, Wan Ting Kong, Olga Zharkova, Sonja C. J. H. Chua, R. Perumal Samy, Lina H. K. Lim

**Affiliations:** ^1^Department of Physiology, NUS Immunology Program, Centre for Life Sciences, Yong Loo Lin School of Medicine, National University of Singapore, Singapore, Singapore; ^2^Department of Anatomy, Yong Loo Lin School of Medicine, National University of Singapore, Singapore, Singapore; ^3^NUS Graduate School for Integrative Sciences and Engineering, National University of Singapore, Singapore, Singapore

**Keywords:** annexins, ANXA1, influenza virus, FPR2, immunomodulatory

## Abstract

Influenza A viruses (IAVs) are important human respiratory pathogens which cause seasonal or periodic endemic infections. IAV can result in severe or fatal clinical complications including pneumonia and respiratory distress syndrome. Treatment of IAV infections is complicated because the virus can evade host immunity through antigenic drifts and antigenic shifts, to establish infections making new treatment options desirable. Annexins (ANXs) are a family of calcium and phospholipid binding proteins with immunomodulatory roles in viral infections, lung injury, and inflammation. A current understanding of the role of ANXs in modulating IAV infection and host responses will enable the future development of more effective antiviral therapies. This review presents a comprehensive understanding of the advances made in the field of ANXs, in particular, ANXA1 and IAV research and highlights the importance of ANXs as a suitable target for IAV therapy.

## Influenza Virus

Influenza viruses (IV) are one of the most common contributors to human respiratory infections in terms of mortality and morbidity. While IV infection occurs across all age groups, individuals who are more susceptible to severe disease conditions and complications consist of infants, elderly, people with chronic diseases or are immunocompromised (Li et al., 2011; WHO, 2018). An estimate of 3–5 million individuals suffer from severe influenza infection annually (WHO, 2018), with 290,000–650,000 resulting in deaths worldwide (Mata et al., 2011; WHO, 2018). Among the four types of influenza viruses (types A, B, C, and D), Influenza A viruses (IAVs) and Influenza B viruses (IBVs) are mainly in circulation and are responsible for seasonal disease epidemics (WHO, 2018). Around 5% of adults and 20% of children worldwide develop symptomatic IAV or IBV each year (Nicholson et al., 2003). In acute infection, patients develop fevers, dry cough, sore throat, fatigue, headache, and upper respiratory tract inflammation (Taubenberger and Morens, 2008). IAV-related complications such as bronchitis, sinus infections, ear infections, and pneumonia may arise, which can result in organ failure and death (CDC, 2018). The recurrence of such seasonal influenza epidemics is primarily due to the constant mutation of the virus, reducing the effectiveness of vaccines while increasing human-human transmission (Petrova and Russell, 2018). Hence, the rat race between vaccine development and virus evolution makes preventive interventions and management of IAV outbreaks clinically challenging.

## Influenza a Viral Proteins

The segmented genome encodes the hemagglutinin (HA), neuraminidase (NA), matrix protein 1, 2, and M2-related protein (M1, M2, and M42, respectively), nuclear export protein (NEP, also known as NS2), nucleoprotein (NP), non-structural protein 1 (NS1), three polymerase acid proteins (PA-X, PA-N115, and PA-N182), two polymerase basic protein 1 proteins (PB1-F2 and PB1-N40) and polymerase basic protein 2 (PB2) (Shaw et al., 2008; Müller et al., 2012; Muramoto et al., 2013). IAV can then be further characterized according to their composition of HA and NA. Table 1 describes the function of the viral proteins encoded by IAV.

**Table 1:** Influenza A viral proteins and their functions.

RNA Segment	Viral protein	Function
1–3	PB1, PB2, PB1/F2, PA	Viral polymerase proteins essential for viral RNA synthesis (Shaw et al., 2008)
4	HA	Recognizes and binds to host sialic acid receptor (Muramoto et al., 2013) Mediates fusion of viral and endosomal membrane (Muramoto et al., 2013) Necessary to prevent premature fusion of virus to endosome thus facilitating timely escape of viral genome into cytoplasm (Ning et al., 2005)
5	NP	Encapsidates viral genome (Fukuyama and Kawaoka, 2011) Interacts with viral polymerase proteins (Fukuyama and Kawaoka, 2011; Liedmann et al., 2014) Interacts with cellular components such as actin, import, and export machinery, etc. (Fukuyama and Kawaoka, 2011)
6	NA	Required for budding of new viral progenies from the surface of infected cells (Zhang et al., 2015) Facilitates viral movement to infected cells by cleaving neuramic acid in respiratory tract mucins (Zhang et al., 2015)
7	M1, M2	M1 – Provides support for virus (Shaw et al., 2008) M2 – facilitates viral uncoating (Shaw et al., 2008)
8	NS1, NS2	NS1 – Inhibits type I Interferon production, and type I IFN pre-mRNA processing (De Clercq, 2006) NS2 – Mediates nuclear export of vRNPs (Shen et al., 2015) NS2 – Regulates of viral RNA transcription (Shen et al., 2015)

## Iav Pathogenesis

The pathogenesis of IAV is dependent on both virus and host factors. Viral HA proteins bind to α2-6 sialylated glycans expressed on human epithelial cells of the upper respiratory tract to facilitate viral entry into the cell. Upon infection, the single-stranded RNA (ssRNA) of IAV is detected by pattern recognition receptors (PRRs) such as endosomal toll-like receptor 7 (TLR7) or cytosolic retinoic acid-inducible gene-I (RIG-I). This induces anti-viral host responses via a series of signaling cascade for the production of type I interferon that limits viral replication. Virus-induced transcription factors such as interferon (IFN) regulatory factor-3 (IRF-3), as well as IRF-7, serve essential roles in the expression of type-I interferons (Ning et al., 2005). Activated PRRs can also induce the production of pro-inflammatory mediators including IL-1, IL-6, IL-18, IL-12, TNF-α, and chemokines such as CCL5 (RANTES), CXCL8, CXCL10, CCL2 (MCP-1) (Fukuyama and Kawaoka, 2011). Macrophages recruited by these chemokines can induce apoptosis in infected alveolar epithelial cells, ultimately leading to lung inflammation and pathogenesis (Fukuyama and Kawaoka, 2011).

Nonetheless, IAV has evolved to overcome these innate responses. The NS1 protein is a multifunctional virulence factor that can repress activation of RIG-I, hence controlling antiviral responses by the host in the cytosol (Mata et al., 2011). Also, viral PB1/PA has been demonstrated to be able to suppress early IFN induction during infection (Liedmann et al., 2014). Nuclear NS1 also interferes with mRNA processing, affecting the proper expression of host antiviral genes (Fukuyama and Kawaoka, 2011). Moreover, due to the extensive genetic and antigenic diversity of IAV and the ability to undergo genetic re-assortment to infect a large host-range, there is a continuous emergence of new influenza viruses that results in periodic pandemics (Taubenberger and Morens, 2008).

## Clinical Implications

At present, there are two fundamental approaches to managing the influenza epidemic: vaccination and, treatment using antivirals. Several influenza vaccines are available with several modes of administration such as forms of nasal sprays, intradermal injections, and flu jet injectors (Zhang et al., 2015). These vaccines often achieve up to 80% efficacy against homologous strains but become less effective in the elderly population and against heterologous IAV strains. However, the high mutability of the IAV and the sizeable antigenic diversity of viral strains emphasize the need to formulate new vaccines annually (Mata et al., 2011). Increasing numbers of antivirals have been approved to facilitate influenza treatment. Amantadine and rimantadine target the IAV M2 proteins, to inhibit uncoating of the virus, while oseltamivir, zanamivir, and peramivir target NA to prevent virus spread (De Clercq, 2006; Shen et al., 2015). However, the emergence of resistant virus strains has rendered M2 inhibitors useless, and there is growing resistance against NA inhibitors such as the H274Y mutation found in the 2009 H1N1 strain that made the virus oseltamivir-resistant (Arias et al., 2009; Shen et al., 2015). Newer drugs have been approved in Japan that target the viral polymerase such as favipiravir, a nucleotide analog; and baloxavir marboxil which is a cap-dependent endonuclease inhibitor (Koszalka et al., 2017; Portsmouth et al., 2017). Novel techniques such as chemical genetic approaches are also employed to identify molecules, such as naphthalimides, which antagonizes NS1 (Mata et al., 2011).

The high mutability and emergence of resistant strains of the virus to available drugs require a focus on host factors that play critical roles in the pathogenesis of IAV. These factors represent attractive options in the development of novel therapeutics to limit viral spread and infectivity.

## Annexins (Anxs)

Annexins are a family of calcium or phospholipid binding molecules found mostly in eukaryotes but absent in yeasts and prokaryotes (Rescher and Gerke, 2004; Lim and Pervaiz, 2007; Mirsaeidi et al., 2016). They are usually located inside the cell of most eukaryotic organisms, however, some ANXs are also secreted. ANXs comprised of conserved Ca^2+^ and membrane-binding modules with conserved four or eight repeat units of amino acids and an N-terminal domain with greatest sequence variation between families (Barton et al., 1991). Therefore, given its diverse Ca^2+^dependent roles at cellular membranes, ANXs can influence other processes such as inflammation, signaling, and proliferation (Nicholson et al., 2003; Taubenberger and Morens, 2008; Li et al., 2011; Mata et al., 2011). The variability in the N-terminal domain sequence most likely specifies the different regulatory properties of the various ANX family members *in vivo* (Gerke and Moss, 2002).

There are more than 100 ANXs identified in different species (Gerke and Moss, 2002). Approximately 12 of these ANXs have been identified in humans and are assigned Annexin A1–A11, and A13 with various tissue distribution and expression (Mirsaeidi et al., 2016). The ability of ANXs to bind to phospholipids in cellular membranes provides a possible link for its role in viral infection especially in the regulation of endocytosis and exocytosis (Enrich et al., 2011). Different families of ANXs have been shown in the literature to possess various functions during influenza virus infection. ANXA1, ANXA2, ANXA5, and ANXA6 amongst others have been found to play roles in inflammation and lung injury mediated by IAV. Here, we will review the functions of these ANXs, in particular, ANXA1, in the pathogenesis of IAV infection.

### Annexin A1

Annexin A1 (ANXA1) also known as lipocortin-I, is a member of the annexin family of proteins formed by 346 amino acids (Gerke and Moss, 2002). It is a 37 kDa protein and possesses the ability to change its structural conformation upon binding to calcium cations (Rosengarth et al., 2001a,b; Gerke et al., 2005). The changes that occur upon a conformational change may reflect a need for interacting with potential receptors and the functions of the proteins. Blood immune cells such as monocytes and granulocytes express high levels of ANXA1 and are the largest source of cellular ANXA1 with lymphocytes possessing moderate expression levels (Perretti et al., 1996b; Oliani et al., 2002).

Interestingly, B cells and platelets do not express ANXA1 (Perretti and Dalli, 2009). ANXA1 may localize in the nucleus, mitochondria, cytoplasm, and on the cellular membrane depending on the stimulus, and this may partially explain the different functions ANXA1 performs under different stimuli (Solito et al., 2006; D’Acunto et al., 2014; Zhao et al., 2016). It exerts its inflammatory actions by being secreted from its cellular sources albeit lacking a signaling peptide (Perretti and Dalli, 2009). These inflammatory actions are mediated via the G protein-coupled receptor, formyl peptide receptor (FPR) (Walther et al., 2000).

### Annexin A1 Signaling

ANXA1 can act endogenously as a signaling molecule, or exogenously, by binding to the FPR1, 2, or 3. Endogenously, ANXA1 regulates MAP kinase phosphorylation (Alldridge and Bryant, 2003), serves as a substrate for EGF tyrosine kinase (Pepinsky and Sinclair, 1986) as well as protein kinases such as PKC (Wang and Creutz, 1992). Furthermore, ANXA1 associates with NEMO, or IKKγ, which in turn regulates NF-κB (Bist et al., 2011). Similarly, ERK and AKT phosphorylation and NF-κB activity were lower in ANXA1 deficient dendritic cells (Huggins et al., 2009). In contrast, studies on fibroblasts have reported that ANXA1 acts as an endogenous inhibitor of MAP kinase activation through the regulation of MAP kinase phosphatase 1 (Yang et al., 2006). ANXA1 can also enhance TGF-β signaling to induce Smad2 phosphorylation, and stimulation of Smad3/4 activity (de Graauw et al., 2010).

The breakthrough in ANXA1 signaling exogenously was shown when ANXA1 and its derived peptide (Ac2-26) were able to reduce the transmigration of neutrophils across endothelial cell monolayers, which was blocked by an FPR antagonist (Walther et al., 2000). After which, several studies have demonstrated that the anti-inflammatory actions of secreted ANXA1 work via FPR1 and FPR2/ALXR (Sugimoto et al., 2016). These receptors can homodimerize or heterodimerize to cause different cellular responses when bound to ANXA1. For instance, ANXA1 and its peptides can activate the p38 MAPK pathway via homodimerized FPR2 receptor (Filep, 2013). However, stimulation of heterodimerized FPR1/FPR2 by ANXA1 peptides results in PMN apoptosis through the activation of caspase 3 (Filep, 2013). Notably, ANXA1, through FPR1, promotes epithelial wound repair through the activation of NADPH oxidase (NOX1) (Leoni et al., 2013). Further, ANXA1 activation of FPR2 results in ERK phosphorylation (Bena et al., 2012) and NF-κB activation (Jia et al., 2013).

### Regulation of Inflammation by ANXA1

With the growing research around this fascinating molecule, many groups have reported the double-edged sword property of ANXA1. Inflammatory conditions can result in the cleavage of full-length ANXA1 (37 kDa) to the cleaved isoform (33 kDa) and the N terminal peptide by proteinase-3 and neutrophil elastase (Rescher et al., 2006; Vong et al., 2007), or by calpain 1 (Williams et al., 2010). Whereas ANXA1 mediates its function via the FPR2, the ANXA1-derived N terminal peptide can activate all three isoforms of FPR (Sugimoto et al., 2016). This activation might explain the differential effects elicited by the whole peptide versus the functions of different N-terminal cleaved portions of the protein which have anti-inflammatory and pro-inflammatory functions (Williams et al., 2010). In addition, full-length ANXA1 induces the homo-dimerization of FPR2/ALXR, which results in p38 MAPK activation (Cooray et al., 2013), while Ac2-26 stimulates hetero-dimerization of FPR1 and FPR2/ALXR with subsequent activation of c-Jun N-terminal kinase (JNK)-signaling, resulting in IL-10 production (Cooray et al., 2013). Furthermore, ANXA1 can be phosphorylated (Pepinsky and Sinclair, 1986; Wang and Creutz, 1992) and sumoylated (Caron et al., 2013), which further confounds its activity.

### ANXA1 as Anti-inflammatory and Pro-resolution Molecule

ANXA1 was first discovered as a phospholipase A2 inhibitor (then known as macrocortin or renocortin) (Cloix et al., 1983) and is well-known for its role as an anti-inflammatory, glucocorticoid-inducible molecule (Perretti and D’Acquisto, 2009). In early studies in numerous animal models of inflammation, administration of ANXA1 or its N-terminal bioactive peptides exerted anti-inflammatory, anti-pyretic, and anti-migratory effects (Cirino et al., 1989, 1993; Errasfa and Russo-Marie, 1989; Davidson et al., 1991; Perretti et al., 1995, 1996a; Zhao et al., 2016). ANXA1 administration induced leukocyte detachment from the inflamed endothelium and reduced leukocyte adhesion and transendothelial migration (Lim et al., 1998). In the absence of ANXA1, mice exhibited aberrant inflammation and resistance to glucocorticoids (Hannon et al., 2003). In addition, ANXA1 activates MAPK phosphatase-1 (MKP-1), which reduces MAP kinase activation, ultimately reducing pro-inflammatory cytokine expression (Yang et al., 2006). ANXA1 has also been shown to enhance apoptotic leukocyte clearance by macrophages without triggering inflammatory mediator production and its subsequent inflammatory cascade (Maderna et al., 2005; Morimoto et al., 2006). In recent years, ANXA1 is shown to redirect M1 macrophages toward anti-inflammatory M2 phenotype, decreasing the expression on inflammatory cytokines like IL-6, IL-1beta, and TNF-alpha (Li et al., 2011; Moraes et al., 2017).

Overall, the anti-inflammatory properties of ANXA1 has been reported in multiple cell types, especially in innate leukocytes like neutrophils and macrophages and highlights the importance of ANXA1 protein and its peptide in regulating host defense as well as participating in resolution of inflammation (Buckley et al., 2014; Serhan, 2014).

### ANXA1 as a Pro-inflammatory Mediator

Conflicting reports on the role of ANXA1 in promoting inflammation have recently emerged. Dendritic cells from ANXA1^-/-^ mice stimulated with LPS, a TLR4 agonist exhibited impaired migration, decreased maturation, and lower pro-inflammatory cytokine production which was associated with impaired NF-κB signaling and a reduced ability to stimulate the adaptive immune response (Huggins et al., 2009). Similarly, macrophages from ANXA1^-/-^ mice stimulated with TLR3 or TLR4 agonists produced less interferon beta (IFNβ) and interferon-stimulated genes, through an association of the C-terminus of ANXA1 with TANK-binding kinase 1, leading to IRF3 phosphorylation (Bist et al., 2013). As mentioned, ANXA1 phosphorylation by protein kinase C (PKC) leads to subsequent translocation of ANXA1 protein to the nucleus, resulting in pro-inflammatory cytokine release (Zhao et al., 2016). Cleavage of ANXA1 with calpain produced peptides of ANXA1 which promoted neutrophil recruitment, which was not observed with Ac-2-26 (Williams et al., 2010).

### Annexin-A1 in Influenza a Virus Infection

It was recently reported that Annexin A1 (ANXA1) enhances IAV infection (Arora et al., 2016). Infection with IAV enhanced ANXA1 expression, and overexpression of ANXA1 in A549 lung epithelial cells enhanced virus replication *in vitro*. ANXA1^-/-^ mice exhibited improved mortality and morbidity when infected with a lethal dose of IAV, with low viral loads in their lungs compared to their wildtype littermates. ANXA1 was found to co-localize with IAV in early and late endosomes, implying that ANXA1 facilitates endosomal trafficking of IAV (Arora et al., 2016). This process is important for subsequent import of vRNP into the nucleus after release from the endosome. Localization of vRNP in the nucleus is essential for transcription of viral mRNA and subsequent translation into an infective virion (Shi et al., 2014). ANXA1 may thus facilitate this process of nuclear vRNP accumulation, resulting in increased viral loads. Furthermore, ANXA1 enhances IAV-induced apoptosis (Arora et al., 2016). The role of virus-induced apoptosis in their replication is still unclear, with a study demonstrating that at the later stages of infection, IAV induces apoptosis to facilitate replication (Zhirnov and Klenk, 2007). Together, ANXA1 appears to have profound effects on IAV replication and pathogenesis, and presents a unique opportunity for control of IAV infection.

### ANXA1 Receptor (FPR2) in IAV Infection

Annexin A1 serves as one of the many ligands for formyl peptide receptor 2 (FPR2) (Maderna et al., 2010; Bena et al., 2012). FPR2, also called Lipoxin A4 receptor, is a G-protein coupled receptor with plasticity in its interacting ligands mediating both a pro-inflammatory and pro-resolution effector response (Walther et al., 2000; Dufton et al., 2010; Cooray et al., 2013). FPR2 is expressed in intestinal epithelial cells, neutrophils, macrophages, and microglial cells (Bonnans et al., 2006; Chiang et al., 2006; Barnig et al., 2013). As a receptor for ANXA1, understanding its role during IAV infection is essential to develop therapeutics targeting the viral disease.

**FIGURE 1:**
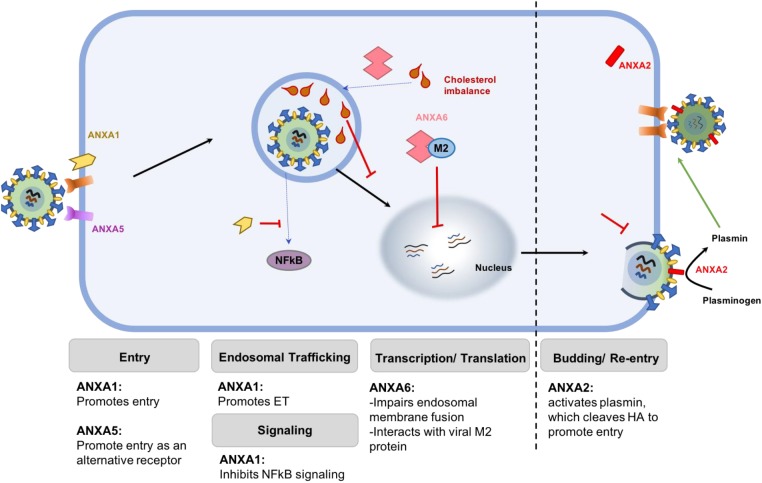
Schematic representation of the differential roles of various ANX family members. ANXA1 promotes viral attachment and endosomal trafficking, thereby enhancing viral replication. ANXA2 incorporates into IAV envelope to convert plasminogen to plasmin, which cleaves HA to facilitate replication. Similarly, ANXA5 acts as an alternative receptor that mediates IAV entry into the cells. Together, these members of the ANX family enhance the replication of virus in infected cells. On the contrary, ANXA6 restricts virus replication by binding to the M2 protein of IAV or by creating an imbalance of cholesterol homeostasis in the late endosome, which prevent viral and endosomal membrane fusion and subsequent export of the vRNP into the nucleus.

One of the earliest reports to implicate FPR2 in IAV infection demonstrated that activation of FPR2 by its ligand WKYMVm-NH2, followed by IAV infection, enhanced pathogenesis and virus replication, while inhibition of FPR2 by its antagonist WRW4, repressed viral replication (Tcherniuk et al., 2016). IAV incorporates ANXA1 into its envelope, suggesting that ANXA1 could potentially activate FPR2 to facilitate virus replication (Figure 1). In this context, it is plausible that secreted ANXA1 can enhance viral replication by activating FPR2, which consequently induces cellular activation of ERK (Pleschka et al., 2001). Blocking FPR2 signaling using its antagonist WRW4 or anti-FPR2 monoclonal antibody, results in the blockage of IAV in endosomes (Rahman et al., 2018). Thus, FPR2 may also contribute to viral replication by facilitating IAV endosomal export into the nucleus.

**FIGURE 2:**
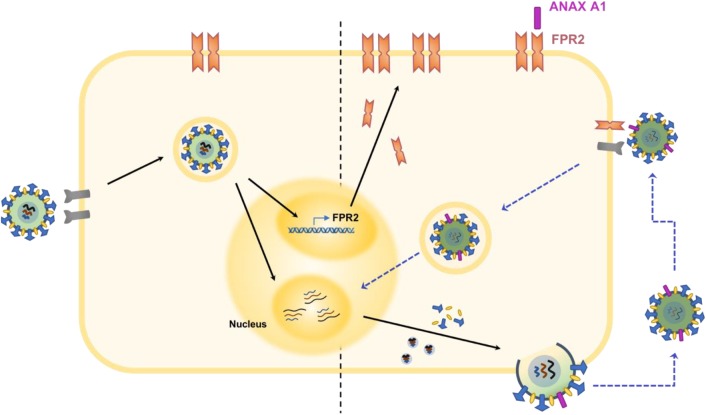
Schematic illustration of the ANXA1 receptor in the pathogenesis of IAV infection. Infection by IAV induces the upregulation of ANXA1 receptor, FPR2 (Ampomah et al., 2017). In addition, ANXA1 is incorporated into the viral envelope during budding of the virus. Increased levels of FPR2 become available for activation by ANXA1 found on viral progenies. This process activates the ERK signaling pathway and ultimately results in increased viral replication (Tcherniuk et al., 2016). Also, FPR2 may enhance endosomal export of vRNP into the nucleus, which enhances viral replication and propagation (Rahman et al., 2018).

Recent studies have shown that IAV infection induces the upregulation of FPR2 in lungs and murine macrophages by stimulating the production of type I interferons (Ampomah et al., 2017). Type I interferon activates the interferon-αβ-receptor and activates signal transducer and activator of transcription factor 3 (STAT3), which induces FPR2 expression. Inhibiting this FPR2 induction pathway reduced viral loads, indicating that upregulation of FPR2 may be essential for virus replication and propagation (Figure 2). ANXA1 may participate in this process by contributing to amplifying type I IFN (Bist et al., 2013), and hence enhancing the upregulation of FPR2.

However, pre-treatment with resolvin D1, a pro-resolution mediator and another agonist for FPR2, reduces the pro-inflammatory effects associated with TLR3 agonist (Poly I:C) treatment in human airway epithelial cells (Hsiao et al., 2014). Although Poly I:C is a viral mimetic and not an actual virus, it is possible that FPR2 activation by Resolvin D1, may be useful in attenuating the inflammatory effects of IAV infection, which happens to be the detrimental side effect of flu infection.

In conclusion, it is of interest to mention that the role of FPR2 in IAV infection remains unclear. This may largely be due to its ability to bind a wide range of ligands which affect inflammation differently. The role of FPR2 in IAV can be studied in the context of enhancing inflammation or resolution. In the future, a murine knockout model will be useful in dissecting the contribution of FPR2 to viral pathogenesis, and how this can be exploited to resolve virus-induced inflammation.

## Other Anxs in Influenza Virus Infection

### Annexin A2

Annexin A2 (ANXA2) is another member of the family of annexins that has been reported to have roles during viral replication. The incorporation of ANXA2 into IAV particles enhance viral replication, a process facilitated by the conversion of plasminogen to plasmin (LeBouder et al., 2008). In addition, it was recently reported that the binding of ANXA2 to highly pathogenic H5N1 influenza virus non-structural protein 1 (NS1) enhances viral replication (Ma et al., 2017).

### Annexin A5

Annexin A5 (ANXA5) was reported in 1994 as a 33 kDa protein that binds 3 different strains of influenza A and one strain of influenza B via a sialic acid-independent mechanism (Otto et al., 1994). It was further shown that ANXA5 aids the entry of influenza viruses into cells, by acting as an alternative receptor for virus entry (Otto et al., 1994; Huang et al., 1996). Blocking of this receptor by adding external anti-serum to cell cultures inhibited virus replication, further highlighting the importance of ANXA5-mediated virus entry in the enhancement of virus replication (Huang et al., 1996). Influenza A virions express host ANXA5 in its membrane, inhibiting interferon gamma signaling and promoting virus replication (Berri et al., 2014).

### Annexin A6

Unlike the previously described members of the ANX family, Annexin A6 (ANXA6) is reported to negatively impact IAV replication during infection. The first evidence on the possible role of ANXA6 in influenza virus infection was its ability to interact with the viral M2 protein to repress replication. IAV M2 protein is essential in the entry and late stage of the viral lifecycle (Shi et al., 2014). Co-expression of a myc-tagged ANXA6 and viral M2, followed by infection provided evidence of a direct interaction between the two molecules (Ma et al., 2012). Moreover, overexpression of ANXA6 reduced virus propagation whereas siRNA-mediated knockdown significantly enhanced virus replication (Ma et al., 2012). ANXA6 may also inhibit IAV replication by increasing late endosomal cholesterol levels (Musiol et al., 2013). By shifting cellular cholesterol pools via ANXA6 to increase late endosomal cholesterol levels, the authors were able to show a decrease in IAV replication and propagation (Musiol et al., 2013). Also, available evidence suggests that the accumulation of cholesterol in the late endosome impairs IAV and endosomal membrane fusion, inhibiting the release of vRNP into the cytoplasm (Kuhnl et al., 2018). Taken together, this suggests that ANXA6 negatively regulates viral replication possibly by interfering with viral M2 function or affecting cellular cholesterol homeostasis.

## Conclusion

The inability of vaccines to induce broad range protection against IAV in circulation, and a possible event of a mutated virus coming into circulation makes the development of therapies that enhance host antiviral mechanisms very desirable. Over the years, considerable efforts have been spent on understanding the roles of various ANX molecules in several infectious diseases, which has been carefully reviewed (Kuehnl et al., 2016; Schloer et al., 2018). To this effect, it can be concluded that ANXs represent very important molecules that are potential targets for further development in the treatment of IAV (Figure 2). As most ANX family members function in the viral entry stage during IAV infection, a broad neutralizing antibody against the ANXA1, ANXA2, and ANXA5 appears to be a suitable option for the treatment of IAV infection in order to lessen the mortality and morbidity associated with the disease. Prevention of the entry phase of the virus infection cycle will thus prevent viral replication and the excessive inflammatory side effects associated with it.

## Author Contributions

PA, WK, OZ, SC, RPS, and LL wrote the manuscript. LL did the final editing of the manuscript.

## Conflict of Interest Statement

The authors declare that the research was conducted in the absence of any commercial or financial relationships that could be construed as a potential conflict of interest.
